# The Association between Branched-Chain Amino Acids (BCAAs) and Cardiometabolic Risk Factors in Middle-Aged Caucasian Women Stratified According to Glycemic Status

**DOI:** 10.3390/nu13103307

**Published:** 2021-09-22

**Authors:** Justyna Kubacka, Paulina Cembrowska, Grazyna Sypniewska, Anna Stefanska

**Affiliations:** Department of Laboratory Medicine, Collegium Medicum, Nicolaus Copernicus University, Sklodowskiej-Curie 9, 85-094 Bydgoszcz, Poland; paulina.cembrowska@wp.pl (P.C.); grazynaodes@interia.pl (G.S.); diag.ka@wp.pl (A.S.)

**Keywords:** branched chain amino acids, cardiometabolic risk factors, glycated hemoglobin, circulating calcium

## Abstract

We examined the glycemic status-stratified relationships between total serum branched-chain amino acid (BCAA) concentrations and cardiometabolic risk factors in middle-aged Caucasian women. The study included 349 women divided into 2 subgroups: a normoglycemic group (NG, *n* = 184) and a dysglycemic group (DG, *n* = 165). Blood samples, anthropometric parameters, and blood pressure were measured. HOMA-IR, albumin-corrected calcium (CCa), and fatty liver index (FLI) were calculated. BCAA concentrations were higher in the women with dysglycemia. BCAAs moderately correlated with BMI and FLI in the NG group and with BMI, FLI, total calcium (TCa), CCa, HbA1c, TG/HDL-C, and HDL-C in the DG group. After adjusting for age and BMI, correlations for TCa, CCa, HbA1c, HDL-C, and TG/HDL-C remained significant. The coexistence of increased BCAAs with dysglycemic status was associated with markedly higher concentrations of TCa, CCa, HbA1c, and TG, which were not observed in the DG women with low level of BCAAs. Multiple regression showed that TCa or CCa, age and BCAAs were significantly associated with HbA1c independently of BMI only in the DG group. We conclude that dysglycemia in particular predisposes women to a significant relationship between total BCAAs and circulating calcium and HbA1c, and that these relationships are independent of BMI and may reflect the pathophysiological calcium-dependent mechanisms connecting BCAAs with metabolic disturbances.

## 1. Introduction

Branched-chain amino acids (BCAAs) possess a branched aliphatic side chain in their structure. This group of amino acids includes leucine, isoleucine, and valine, which are not only components of proteins, but also regulate many physiological processes in the human body [1]. BCAAs are mainly found in muscle tissue, constituting 14–18% of all amino acids that build the proteins of this tissue. They belong to the group of exogenous amino acids that must be supplied to the body through the diet [2]. BCAAs are present in raw materials and food products containing both vegetable and animal proteins. The main food sources with the highest BCAA contents are meat, fish, grains, dairy products, vegetables, and eggs [2,3]. Nevertheless, some reports suggest that a small number of these amino acids can also be synthesized by the gut microbiome [4,5]. In addition to the well-known role of BCAAs in the metabolism of muscle proteins, there has been an increasing interest in the role of BCAAs in cardiometabolic diseases, especially in the context of obesity, insulin resistance, and diabetes. It has been found that elevated levels of circulating BCAAs correlate with obesity, and an increased risk of insulin resistance (IR), and type 2 diabetes mellitus (T2DM) in both human and animal models [6,7,8]. It was also found that BCAAs contribute to the development of obesity-associated insulin resistance [8]. An increased concentration of these amino acids may even appear several years before the development of full-blown type 2 diabetes [9]. However, whether BCAAs are simply markers of insulin resistance or directly contribute to insulin resistance remains uncertain. It has been suggested that circulating BCAAs increase negative feedback (by activating the target mammalian rapamycin) to the insulin receptor 1 substrate, which promotes insulin resistance and impairs glucose metabolism [8]. The relationship between BCAAs and other cardiometabolic risk factors is less well known. Yang et al. found that BCAA concentrations are positively associated with the risk factors of coronary artery disease (CAD), e.g., carotid intima-media thickness (cIMT), BMI, blood pressure, fasting blood glucose, TG, apoB, apoB/apoAI ratio, and CRP, and negatively with HDL-C [10]. A recent study has suggested that BCAAs could present lipid-related cardiovascular (CVD) risks to some extent by affecting lipid metabolism in the Chinese population [11]. It was found that the association between BCAAs and CVD risk is much more pronounced in women who developed T2DM prior to a CVD event and the authors conclude that impaired BCAA metabolism may represent a pathway underlying the pathophysiology that links the risks of T2D and CVD [12]. Mels et al. also observed that BCAAs are independently related to blood pressure and cIMT in individuals with high HbA1c levels, which may suggest that BCAAs play a role in cardiovascular health, especially in the conditions of hyperglycemia [13].

Because of previous evidence suggesting that the glycemic status of subjects may be related with the cardiometabolic effects of BCAAs, we aim to evaluate the association of total BCAA concentrations with selected cardiometabolic risk factors in women with normoglycemic and dysglycemic status. We focused on the conventional risk factors for CVD, such as hyperlipidemia, hypertension, increased inflammatory state, fatty liver, and decreased estimated glomerular filtration rate (eGFR). As calcium homeostasis plays a role in glucose and amino acid metabolism [14,15], as well as being recognized as a cardiometabolic risk factor [16], we also examined the relationship between circulating calcium concentration and BCAAs in relation to glycemic status. We studied a group of middle-aged women because this group is especially exposed to an increased risk of developing an unfavorable cardiometabolic profile. Moreover, some earlier studies suggest that the relationship between BCAAs and glycemic status is weaker in women than in men [17,18]. Therefore, we hypothesized that a dysglycemic state is associated with the cardiometabolic effect of BCAAs in middle-aged women.

## 2. Materials and Methods

### 2.1. Participants

The study included 349 Caucasian women. The women were selected from a group of women who had previously participated in a cardiometabolic risk factors study at the Department of Laboratory Medicine of the Nicolaus Copernicus University in Bydgoszcz, Poland between 2016 and 2019. The exclusion criteria were as follows: history of CVD, diabetes mellitus type 1, familial hypercholesterolemia, severe or moderate chronic kidney disease (CKD), and thyroid and parathyroid disorders. All the women had serum CRP < 10 mg/L, and thyroid stimulating hormone (TSH) < 4.94 μIU/mL, TC < 8 mmol/L, GFR > 60 mL/min/1.73 m^2^. Age, smoking habits and physical activity, menopausal status, medical history, and medications were investigated using a medical questionnaire. Height (cm), weight (kg), and waist circumferences (WC, cm) were measured using standard methods. Systolic and diastolic blood pressures were measured twice in accordance with standard procedures, in the sitting position after at least 10 min of rest. Postmenopausal status was defined as the permanent cessation of menstruation for at least 12 months. Smoking status was classified as current smoker, ex-smoker, and nonsmoker. Physical activity was classified as never (0 point), sporadically (1 point), more than once a month (2 points), once a week (3 points), 3 to 4 times a week (4 points), and every day (5 points). Hyperlipidemia was defined as triglycerides ≥ 150 mg/dL (1.7 mmol/L) total cholesterol ≥ 190 mg/dL (5.0 mmol/L), LDL-cholesterol ≥ 115 mg/dL (3.0 mmol/L) and HDL-cholesterol ≤45 mg/dL (1.2 mmol/L) or use of lipid-lowering medications due to a previous diagnosis. Hypertension was defined as ≥140/90 mmHg or use of hypertension treatment [13]. Type 2 diabetes was diagnosed according to a fasting glucose level in plasma ≥ 126 mg/dL (≥7.0 mmol/L) repeated on two consecutive days, or HbA1c above or equal to 6.5% (48 mmol/mol) or self-reported, physician-diagnosed diabetes and the use of glucose-lowering medications for diabetes. Impaired fasting glucose (prediabetes) was indicated by a fasting glucose level in plasma of 5.6–6.9 mmol/L (100–125 mg/dL) and a normal fasting glucose of <5.6 mmol/L (100 mg/dL) [19]. An insulin-resistant state was diagnosed in women with HOMA-IR > 2.3 [20]. The median cutoff value for BCAAs was used [475.0 µmol/L (422.8–566.4); *n* = 349]. The 75th percentile cutoff values for total calcium (TCa), albumin-corrected calcium (CCa) and glycated hemoglobin (HbA1c) were used [2.36 mmol/L (2.27–2.45); 2.26 mmol/L (2.20–2.38); 37.0 mmol/mol (33.0–39.0) *n* = 349, respectively].

The study participants continued their normal omnivorous diet regimen. None of them followed an elimination or high-protein diet. Dietary supplements, including BCAAs, calcium, vitamin D and protein supplements (e.g., whey, casein), were not used either.

The women were divided into 2 groups. A normoglycemic group (NG, *n* = 184): women with a normal fasting glucose, without a history of diabetes, and with HOMA-IR < 2.3. A dysglycemic group (DG, *n* = 165): women with at least one of the following criteria: diabetes, prediabetes, HOMA-IR ≥ 2.3.

### 2.2. Blood Sampling & Laboratory Analyses

Blood samples were collected in the early morning (7.00–9.00 a.m.) after an overnight fast (12 h). The serum was assayed for TC, LDL-C HDL-C, TG, creatinine, CRP, gamma glutamyltransferase (GGT), total calcium, and albumin, and the plasma was measured for glucose (Horiba ABX Pentra 400), and TSH (ARCHITECT ci8200, Abbott Diagnostics). The serum insulin concentration was determined using a sandwich ELISA method: Insulin: DRG MedTek, with intra-assay precision of 2.8–4.0%, and inter-assay precision of 2.6–3.6%; BCAAs were assayed using a colorimetric enzyme test (Kit Immundiagnostik AG, Bensheim, Germany for the in vitro determination of total BCAAs as a sum of L-leucine, L-isoleucine and L-valine in serum) with intra-assay precision of 4.2–5.5%, and inter-assay precision of 3.7–8.9%. The EDTA blood samples were analyzed for glycated hemoglobin (HbA1c) using a Bio-Rad VARIANT II turbo (HPLC). The HOMA-IR value was calculated by dividing the fasting insulin concentration (mU/L) and the glucose concentration (mmol/L) by 22.5 [20]. The estimated glomerular filtration rate (eGFR) was calculated using the CKD-EPI creatinine equation. A normal eGFR was defined as greater than 90 mL/min/1.73 m^2^ [21]. The fatty liver index (FLI) was calculated using the following formula:

FLI = (e0.953 × loge(triglycerides) + 0.139 × BMI + 0.718 × loge(GGT) + 0.053 × waist circumference − 15.745)/(1 + e0.953 × loge(triglycerides) + 0.139 × BMI + 0.718 × loge(GGT) + 0.053 × waist circumference − 15.745) × 100.

High risk for non-alcoholic fatty liver disease (NAFLD) was defined as FLI ≥ 60 [22].

Albumin-corrected calcium was calculated using the formula: corrected calcium (mmol/L) = serum total calcium + 0.02* (40 − serum albumin (g/L) [23].

The study protocol was approved by the Bioethics Committee at the Nicolaus Copernicus University in Torun, Collegium Medicum in Bydgoszcz, and written informed consent was obtained from all the women.

### 2.3. Statistical Analysis

The data were presented as means ± standard deviation SD (Gaussian distribution) or medians and 25th and 75th percentiles (non-Gaussian distribution). The Shapiro–Wilk test was applied to test the Gaussianity. The variables were compared using the Student’s *t*-test (Gaussian) or the Mann–Whitney *U* test (non-Gaussian). To test for the significance of difference between two percentages or correlation coefficients we used the chi-square (Fisher exact) test. Significant differences between groups were also tested with the analysis of covariance (ANCOVA) with adjustment for BMI, age, and menopausal status. Parameters with non-Gaussian distribution were normalized by natural log transformation. Pearson and partial correlation coefficients were computed to assess the associations between BCAAs and various parameters (log-transformed variables). In multiple linear regression analysis, BMI, age, BCAAs, TCa or CCa were assayed as independent variables and HbA1c as a dependent variable. We applied two-way ANOVA models to evaluate the association between values of the dependent variables (cardiometabolic risk factors) and the qualitative factors: NG or DG group and BCAA concentrations above or below their median. Logistic regression was applied to examine the associations of BCAA concentration with cardiometabolic risk factors. In all the logistic models, BCAAs were included, and odds ratios (ORs) were calculated for a 10 unit increase in BCAAs. Additional logistic regression models were adjusted for age and BMI. The significance of BCAA coefficients in the logistic models was tested using Wald chi-squared statistics. The goodness of fit of the models was evaluated using the Hosmer and Lemeshow chi-square test. Additionally, ROC curves were constructed for single laboratory parameters and the area under each curve was calculated with a 95% confidence interval (AUC, 95% CI; thresholds with sensitivity and specificity). A comparison of independent ROC curves was performed. During sample size determination, a significance level of 0.05 was applied for parametric two-tailed t-Student tests with a power level of 0.9. For nonparametric Mann–Whitney tests, the minimum sample size required was increased by 15%. According to the pilot study, BCAA concentrations for the first 63 women in the DG group and for the first 59 in the NG group were 556 ± 93 and 452 ± 80 µmol/L, respectively. Based on these results, we calculated that enrolment of 184 women in the NG group and 165 women in the DG group would provide a power of 100% to show a significant difference in BCAA concentrations. We decided to obtain such a high power to be able to perform credible multivariate analyses. The level of statistical significance was set as 0.05 (Statistica 13.3, StatSoft or MedCalc statistical software).

## 3. Results

The basal characteristics of the groups are shown in Table 1. BCAA concentrations were significantly higher in the women with dysglycemia in comparison to those with normoglycemia. This difference was still significant after adjusting for BMI, age, and menopausal status (Table 1).

As shown in Table 2, all parameters were statistically significantly correlated (*p* < 0.05) with BCAA concentrations, except the DBP values in all the women. After adjustment for BMI and age, the correlations with LDL-C, TC/HDL-C, eGFR, FLI, CRP, and SBP became statistically insignificant. After considering the glycemic status, BCAA concentration was moderately (0.4 ≤ r < 0.6) correlated with BMI and FLI in the NG group and with BMI, FLI, TCa, CCa, HbA1c, and TG/HDL-C in the DG group in unadjusted models. The unadjusted and adjusted correlation coefficients for TCa, CCa, HbA1c, HOMA-IR, and glucose were significantly higher in the DG group in comparison to the NG group (*p* < 0.0001; *p* = 0.002; *p* = 0.004, *p* = 0.05, *p* = 0.05 respectively; Table 2). Because BCAA concentrations are very strongly associated with BMI and tend to increase with age, we adjusted all the correlations to BMI and age. After adjustment, we observed that most of the correlations became statistically insignificant or were still significant but with very weak correlation coefficients (r < 0.2), especially in the NG group. Adjusted correlation coefficients were classified as weak (0.2 ≤ r < 0.4) and were still statistically significant for eGFR (NG group), TCa, CCA, HbA1c, glucose, HDL-C, and TG/HDL-C (DG group) (Table 2).

**Figure 1:**
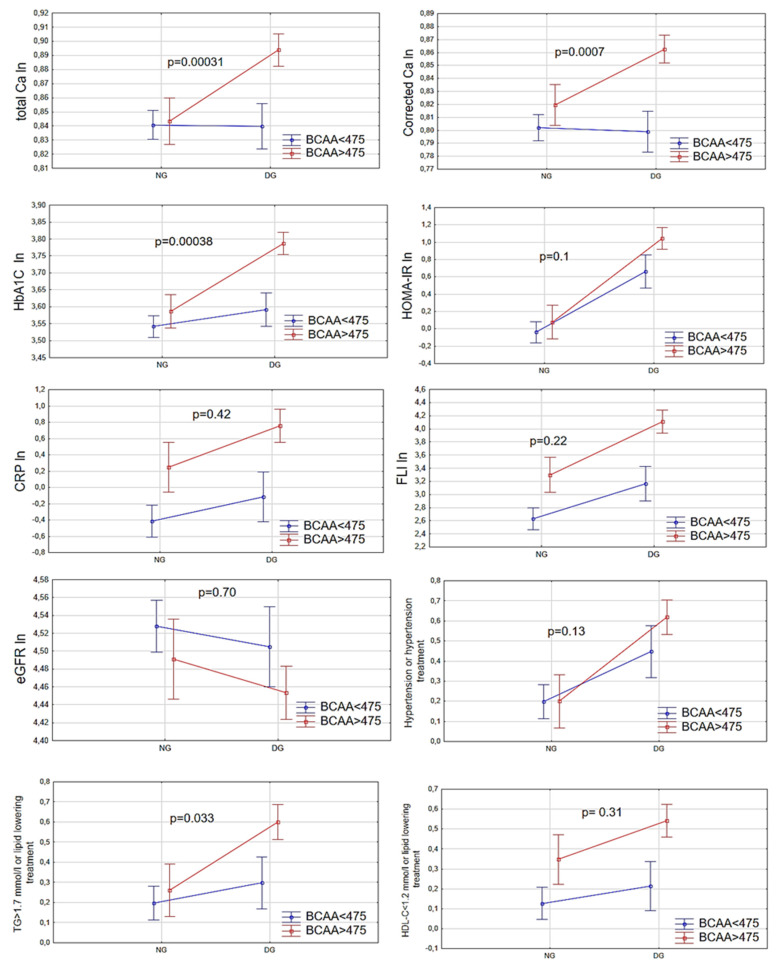
Results of cardiometabolic risk factors in relation to glycemic status (NG, DG) and BCAA concentrations above or below their median (475 µmol/L) in the women. Two-way ANOVA; the glycemic status and BCAA subgroups as the qualitative factors, and total Ca, corrected Ca, HbA1c, HOMA-IR, CRP, FLI, eGFR, hypertension or hypertension therapy, and dyslipidemia (HDL < 1.2 mmol/L, TG > 1.7 mmol/L or lipid-lowering treatment) as dependent variables. NG—normoglycemic group, DG—dysglycemic group.

We applied two-way ANOVA models (Figure 1) to evaluate the association between values of the cardiometabolic risk factors, e.g., corrected Ca, total Ca, and CRP etc. with glycemic status (NG or DG group) and BCAA concentrations above or below their median (475 µmol/L). This analysis aims to examine how the coexistence of NG or DG status with an elevated BCAA concentration affects the values of cardiometabolic risk factors. In summary of the comparisons of the NG and DG women after defining subgroups according to the values of BCAAs, we observed that the differences in values of TCa, CCa, and HbA1c, and the occurrence of hypertriglyceridemia were statistically significantly higher between the NG and DG groups when the women had BCAA concentrations above 475 µmol/L in comparison to women with BCAA concentrations below 475 µmol/L. In other words, these results may suggest that the coexistence of increased BCAA values (above the median) with dysglycemic status is associated with markedly higher concentrations of TCa, CCa, HbA1c, and TG (p for difference between NG and DG in women with BCAAs > 475 µmol/L: all *p* < 0.0001), which was not observed in women with BCAA values below the median (*p* for difference between NG and DG in women with BCAAs < 475 µmol/L: 0.90; 0.71; 0.01; 0.16; respectively), while the increases in the values of HOMA-IR, CRP, and FLI, the occurrence of hypertension or HDL-C < 45mmol/L or their treatment, or a decrease in eGFR in the DG group were not statistically different between BCAA subgroups (Figure 1).

To assess the association between BCAAs and cardiometabolic risk factors in total, and in both NG and DG groups separately, we performed logistic regression- and ROC curve- analyses. As shown in Table 3, the BCAA serum levels were significantly associated with the probability of all cardiometabolic risk factors in all groups, except TC/HDL-C > 4.5 (DG group) and TC/HDL-C > 4.5, TG > 1.7, HbA1c > 39 and hypertension or hypertension treatment (NG group). In the unadjusted models, each 10 µmol/L increase in BCAA concentrations increased the probability of cardiometabolic risk factors between 2% for TC/HDL-C > 4.5 in the DG group and 19% for FLI > 60 in the NG group. We observed higher associations of BCAA concentration with CCa > 2.38 mmol/L, TCa > 2.45 mmol/L, and HbA1c > 39 mmol/mol in the DG group in comparison to the NG group in the crude models, while the associations between BCAAs and FLI > 60 or CRP > 3.0 mg/L were stronger in the NG group. After adjusting for BMI and age, models for HbA1c > 39 mmol/mol (in total and DG group), CCa > 2.38 mmol/L (in total and DG group), TCa > 2.45 mmol/L (in total and DG group), eGFR < 90 (in total and NG group), HDL < 1.2 mmol/L (in total and DG group), and TG > 1.7 mmol/L (in total) were still statistically significant (Table 3). After additional adjustment for glycemic status (in total group), models for CCa > 2.38 mmol/L (OR 95%CI 1.07 (1.03–1.12)), TCa > 2.45 mmol/L (OR 95%CI 1.05 (1.01–1.09)), HbA1c > 39 mmol/mol (OR 95%CI 1.04 (1.0–1.09)) and eGFR < 90 (OR 95%CI 1.04 (1.0–1.07)) were still statistically significant (*p* < 0.05).

ROC curve analyses revealed that BCAAs had good diagnostic accuracy for prediction of the occurrence of CCa > 2.38 mmol/L (DG; AUC (95CI) 0.84 (0.76–0.89); sensitivity 89% and specificity 73%) and FLI > 60 (NG; AUC (95CI) 0.85 (0.79–0.91); sensitivity 81% and specificity 75%), according to the AUC value in the ROC analysis, and fair diagnostic accuracy for the prediction of the occurrence of TCa > 2.45 mmol/L (DG), HbA1c > 39 mmol/mol (DG), FLI > 60 (NG), CRP > 3.0 mg/dL (NG) (Table 4).

As our previous results showed that dysglycemic status has the biggest impact on the relationship between BCAAs and serum calcium or HbA1c concentrations, we decided to evaluate the additional correlation and multiple regression analyses between these parameters. We found that HbA1c did not correlate significantly with TCa (r = 0.09 *p* = 0.25) or CCa concentrations (r = 0.14, *p* = 0.06) in the normoglycemic group, while we observed a significant relationship between HbA1c and TCa (r = 0.40, *p* < 0.0001) or CCa (r = 0.46, *p* < 0.0001) in the dysglycemic group.

In order to examine which parameters are most strongly related to values of HbA1c, we analyzed the multiple regression between HbA1c (dependent variable) and BCAAs, serum calcium (CCa or TCa), BMI and age (independent variables) in the DG group. We observed that BCAAs, TCa or CCa, and age were significantly associated with HbA1c concentration independently of BMI values (BMI was insignificant in these models) in the dysglycemic group (model with TCa: R2 = 0.28 *p* < 0.0001; TCa beta 0.21, *p* = 0.017; BCAAs beta = 0.3, *p* = 0.0008; age beta = 0.18, *p* = 0.03) or (model with CCa: R2 = 0.28 *p* < 0.0001; CCa beta = 0.29 *p* = 0.0009; BCAAs beta = 0.23 *p* = 0.005; age beta = 0.16, *p* = 0.03).

## 4. Discussion

The main finding of this study is that dysglycemic status predisposed individuals to a significant positive association between concentration of total BCAAs and circulating calcium, HbA1c, glucose, and HOMA-IR, and these relationships were independent of BMI and age. We also found significant correlations with CRP, HDL-C, TG, FLI, and eGFR, but these relationships were partially explained by BMI and age, except for HDL-C, which correlated with BCAAs independent of BMI and age. The relationships between BCAA concentrations and conventional cardiometabolic risk factors like dysglycemia, dyslipidemia, fatty liver disease, and inflammation have been shown by other authors. However, to the best of our knowledge, the relationship between concentration of BCAAs and serum calcium in combination with glycemic status has not been studied so far.

### 4.1. The Relationship of BCAAs with the Assessments of Glycemic Status

In this study, the total BCAA concentrations were significantly higher in the DG women even after adjusting for BMI and age, which suggests that dysglycemic status per se was associated with higher concentrations of BCAAs. The relationship between BCAAs and insulin-resistance and diabetes is well known [8,17,24,25,26,27]. We also observed statistically significant correlations between BCAAs and glucose, HbA1c and HOMA-IR values in all the studied women. Some earlier studies found that the concentrations of BCAAs are lower in women than in men and the relationship between glycemic status and insulin resistance is weaker in women in comparison to men, especially at a young age and with a normoglycemic status [17,18]. On the other hand, the positive relationship between BCAAs and HOMA-IR, regardless of gender, age, T2DM, or BMI, has been shown by other authors [24,25]. In this study, we found that the relationship of total BCAAs to HOMA-IR was significantly weaker in normoglycemic middle-aged women in comparison to dysglycemic women, and this relationship persisted after adjustment for BMI and age in the dysglycemic state. Contrary to our correlation results, Wiklund et al. found that normoglycemic women with high HOMA-IR (above median HOMA-IR > 1.56) had higher serum BCAA concentrations in comparison to low HOMA-IR normoglycemic women and the authors concluded that this insulin resistance was associated with increased serum BCAA levels in the normoglycemic state [27]. This difference may be partially explained by the fact that our normoglycemic women had the more efficient glucose metabolism (a lower median of HOMA-IR), which could weaken the pathophysiological relationships between BCAAs and insulin resistance. Moreover, the results of two-way ANOVA showed that the NG women with high BCAA concentrations had only slightly higher HOMA-IR, which may suggest that in the normoglycemic condition without insulin resistance (HOMA-IR < 2.3) increased BCAA concentrations have a weaker association with glucose metabolism.

In this study, the relationship between BCAAs and HbA1c was stronger in the dysglycemic group of women than in the normoglycemic one. The probability of a high HbA1c with each 10 µmol/L increase in BCAA concentration was about 4 times lower in the NG group in comparison to the DG group (3% vs. 11%). Moreover, the coexistence of increased BCAA values with dysglycemic status was associated with markedly higher concentrations of HbA1c. Although previous studies have extensively described the relationship between BCAAs and HOMA-IR, so far only individual, isolated studies have also considered the relationship between BCAAs and HbA1c. Fiehn et al. observed that concentrations of leucine and valine increased with an increase in HbA1c in obese African American women [28]. In 2017, Barceló et al. observed that isoleucine levels were associated with HbA1c even more strongly than with HOMA-IR in subjects with morbid obesity and sleep apnea [29]. Mels et al. observed that, in individuals with impaired glucose metabolism and elevated levels of glycated hemoglobin (HbA1c > 5.6%), BCAA concentrations were significantly higher than in the group with low HbA1c levels [13]. Therefore, our results are consistent with other observations which have been performed in obese and dysglycemic women. To the best of our knowledge, the relationship between BCAA and HbA1c values in normoglycemic Caucasian women without insulin resistance has not been studied so far. However, our results for HbA1c seem to be consistent with our results for HOMA-IR.

### 4.2. Potential Pathophysiology Linked BCAAs with Dysglycemia

Various factors are involved in the regulation of circulating concentrations of BCAAs. The most important of these are dietary, genetic, and metabolic factors. With regard to dietary factors, we know that dietary proteins and amino acids are important modulators of glucose metabolism and insulin sensitivity [30]. The consumption of certain protein products, such as increased consumption of processed red meat in particular, which is also a source of BCAAs, has been linked to biomarkers of inflammation, the development of type 2 diabetes, and cardiovascular disease [31,32]. It is worth noting, however, that among the total pool of amino acids contained in red meat, BCAAs do not exceed 16%; the rest are other essential and non-essential amino acids, especially large neutral amino acids, the intake of which, along with the daily diet, significantly exceeds the intake of BCAAs [33]. It is of note that amino acids other than BCAAs that are found in food proteins may also be connected to insulin secretion or insulin resistance as well as having a potential influence on BCAA metabolism and utilization. For this reason, it would be important to analyze the plasma concentrations of other amino acids, e.g., glutamine, to understand this complex issue [34,35,36]. Our women continued their normal omnivorous diet regimen. None of them followed an elimination or high protein diet or took BCAA protein supplements. There are also reports suggest that protein intake and total calories do not significantly affect the relationship between BCAAs and insulin resistance or diabetes risk [9,37]. In the longitudinal Nurses’ Health Study, high total dietary BCAA intake was associated with a risk of T2DM only among women with high plasma BCAA concentrations at baseline, which may suggest that factors other than dietary intake may determine plasma BCAA levels [38]. It is a very controversial issue whether BCAAs are a cause or consequence of insulin resistance. There is much evidence supporting the hypothesis that an increase in plasma BCAAs may be a consequence of insulin resistance. It is well-documented that obesity and insulin-resistance are associated with reduced catabolism of BCAAs caused by lowered activity of the branched-chain α-keto-acid dehydrogenase complex (BCKDC), which results in accumulation of BCAAs and branched-chain-ketoacids [39,40]. In 2013, Lackey et al. found that metabolically unhealthy obese humans had lower BCAA catabolic enzyme expression in white adipose tissue (WAT) in comparison to obese healthy subjects and improvement in glucose utilization in adipocytes was associated with higher expression of BCAA catabolic enzymes in adipocytes and WAT [41]. A recently published Mendelian randomization report also supports this hypothesis, indicating that HOMA-IR is causally related to higher circulating fasting BCAA levels according to genetic risk score analysis [42]. It was also found that therapies with BCAAs did not raise the concentrations of glucose in patients with liver cirrhosis [43,44] and prolonged essential amino acid supplementation (with 62% BCAAs) did not decrease insulin sensitivity in healthy older adults with moderate protein intake [45]. Moreover, BCAA levels were decreased with improvement in HbA1c values after glucose-lowering therapies in T2DM patients [46]. On the other hand, other studies suggest that accumulation of BCAAs may have a negative effect on insulin sensitivity and can play a causal role in the development of insulin resistance and T2DM. One possible mechanism is that BCAAs promote insulin resistance by activating the mammalian target of the rapamycin 1 complex (mTORC1) and ribosomal protein S6 kinase 1 (S6K1). High concentrations of BCAAs activate the mTORC1/S6K1 kinase pathway, resulting in insulin resistance through the phosphorylation of insulin receptor substrate 1 (IRS-1), leading to blocked insulin signaling [47,48]. Moreover, Saha et al. observed that incubation of rat skeletal muscle with moderately elevated concentrations of leucine or glucose suppresses 5’AMP-activated protein kinase (AMPK) activation and concomitantly increases mTORC1/S6K1 signaling and protein synthesis and leads to insulin resistance [49]. The results of our two-way ANOVA suggest that DG status is mainly associated with an increase in HOMA-IR values with a lower impact of high BCAAs, while an increase in the concentration of HbA1c is statistically stronger when dysglycemia coexists with high BCAA values. We hypothesize that this may be explained by the fact that chronic exposure to moderately elevated BCAA values added to hyperglycemia or pro-inflammatory conditions could decrease the threshold of mTOR phosphorylation and increase reactive oxygen species (ROS) formation [50]. The accumulation of ROS results in the induction of a glycoxidation reaction, which may lead to the elevated endogenous production of HbA1c and advanced glycation end-products (AGEs) [51]. We also observed that, even after adjustment for age, BMI and glycemic status, the association between BCAAs and HbA1c persisted, which may suggest that different mechanisms regulate this relationship.

### 4.3. The Association between BCAAs and Serum Calcium Concentration in Relation to Glycemic Status

As our results showed that dysglycemic status has the biggest impact on the relationship between BCAAs and serum calcium or HbA1c concentrations, we decided to evaluate more precisely the relationship between these parameters. We found higher concentrations of serum calcium in the DG group in comparison to the NG group. HbA1c concentrations were significantly positively related with serum calcium concentrations independently of BMI and age in the dysglycemic women, while we did not observe such a relationship in the normoglycemic group. The concentration of serum calcium may be used as a cardiometabolic risk factor because many studies have confirmed the positive association of circulating calcium levels with a higher risk of vascular disease and death, which was presented in a meta-analysis of Reid et al. However, as the authors of this meta-analysis emphasize, the determination of causality in this relationship is difficult [16]. Many studies have provided evidence that higher circulating calcium levels are associated with an increased risk of T2DM, which was recently summarized in a meta-analysis of Zhu et al. [52]. It has also been found that elevated serum calcium concentrations are associated with insulin resistance and impaired glucose tolerance [53,54,55]. But the results of some case–control and cross-sectional studies do not support these observations [56,57]. Additionally, the data on the correlation between serum calcium concentration and HbA1c values are inconsistent. Akter et al. found that serum calcium levels are positively associated with HbA1c among apparently healthy Japanese adults [58]. In contrast, Hassan et al. and Marshnil et al. observed a negative relationship between serum calcium and HbA1c in T2DM patients [59,60]. These differences may be partially explained by the use of different calcium measurements or heterogeneous study populations. The concentration of serum calcium may be analyzed as total calcium, albumin-corrected calcium, or ionized calcium. The measurement of ionized calcium seems to be the best form because it represents the physiologically active proportion of calcium in the serum, but the method of its measurement is difficult and for that reason it is not routinely used, even in research studies [61]. In this study, we analyzed total calcium and albumin-corrected calcium. The adjustment of calcium concentration for albumin is advisable because approximately 40% of Ca in the serum is bound to albumin [62]. Nevertheless, our results for total calcium and albumin-corrected calcium are generally consistent, although the correlation coefficient values for total calcium and BCAAs were weaker than for albumin-corrected calcium, especially in the normoglycemic women. Differences were also observed among both sexes and ethnic groups [63,64]. The pathophysiological connection between serum calcium concentration and glucose metabolism is mainly explained by the function of the calcium-sensing receptor (CaR), which is expressed in pancreatic islets of Langerhans [65]. Glucose-dependent insulin secretion from the pancreatic β-cells is a process that is regulated by the calcium-dependent signaling pathway [14,66,67] and a high calcium concentration may induce β-cell dysfunction [63]. Moreover, it was observed that calcium status impacts insulin sensitivity and glucose transport in adipocytes and skeletal muscle through regulating the glucose transporter type 4 (GLUT4) expression [68,69].

As mentioned above, the relationships between insulin-resistance or diabetic state and BCAAs or serum calcium have been presented by other authors but, to the best of our knowledge, there is no evidence concerning the relationship between serum calcium and BCAA concentrations in relation to the assessment of glycemic status.

Our results show that the coexistence of increased BCAA values with dysglycemic status is associated with markedly higher concentrations of serum calcium, which is not observed in dysglycemic women with BCAA values below the median. These results may suggest that the coexistence of elevated BCAA levels with a dysglycemic state is closely related with circulating calcium concentration. In the normoglycemic women, we did not observe such differences between BCAA subgroups. Moreover, our multiple regression analysis showed a positive association between HbA1c and serum calcium and BCAAs independently of BMI only in the dysglycemic women.

### 4.4. Potential Pathophysiology Linked BCAAs Metabolism with Circulating Calcium Concentration

We hypothesize that the mTOR signaling pathway may have linked the BCAAs to calcium and glucose metabolism. However, the results of our study are not able to establish a cause-and-effect relationship between these factors. As mentioned above, high concentrations of BCAAs may activate the mTORC1/S6K1 kinase pathway, resulting in insulin resistance. It has also previously been observed that calcium ions (Ca^2+^) are required for mTORC1 and S6K1 activation in response to amino acids, especially leucine. Mercan et al. showed that activation of S6K1 by leucine requires the mobilization of intracellular calcium (Ca^2+^), which is mediated by the protein tyrosine phosphatase SHP-2 in an inositol-1,4,5-trisphosphate-dependent manner in skeletal myoblasts. Interestingly, the increase in Ca^2+^ is specific to leucine since isoleucine did not induce a Ca^2+^ response under similar conditions [70]. However, a thorough understanding of how amino acids control Ca^2+^-mediated activation of S6K1 has yet to be fully attained. Therefore, our findings may suggest that a dysglycemic state with elevated BCAAs and serum calcium concentrations may reflect the increased mTORC1 activation. Moreover, it was also found that prolonged culture with leucine upregulates ATP synthase, glucokinase, and cytosolic Ca^2+^ and results in glucose-induced insulin secretion in rat β-cell islets [71]. Glucose also inhibits leucine’s effects on insulin secretion via allosteric inhibition of glutamate dehydrogenase [72]. Thus, it seems that leucine and glucose in calcium-dependent processes may regulate metabolism using different mechanisms which are not fully understood. In this study, we measured serum calcium, but most of the above-mentioned reactions involve cytosolic calcium. There is some evidence which links serum calcium to its cytosolic level. The elevated concentration of serum calcium reflects its extracellular level, which may be related with impairment of voltage-gated calcium channels, which are necessary for insulin secretion [73]. Moreover, Aoki et al. observed that an increased serum calcium concentration led to calcium influx into arterial smooth muscle, which increases cytosolic calcium [74].

### 4.5. The Relationship of BCAAs with Other Conventional Cardiometabolic Risk Factors

In our study, we also observed a statistically significant negative correlation between total BCAAs and HDL-C concentration and positive correlations between BCAAs and TG or TG / HDL-C values in all the women even after adjustment for age and BMI. Additionally, our results suggest that the relationship between total BCAAs and HDL-C is independent of BMI, age, and glycemic status, while the relationship with TG is stronger in dysglycemic conditions and is mainly explained by obesity. Moreover, our two-way ANOVA showed that the coexistence of elevated BCAA values with dysglycemia particularly predisposes women to significantly higher concentrations of TG. Our findings are partially consistent with previous reports. Two prospective studies showed that increased serum BCAA concentrations are associated with an increased risk of elevated TG concentration in European and Japanese populations [75,76]. Another Chinese study showed that total BCAA concentrations are associated not only with elevated TG levels but also with lowered HDL-C levels, independent of fasting blood glucose, and the ORs for reduced HDL-C and raised TG were higher in a high HbA1c group in comparison to a low HbA1c group [11]. In 2019, Fukushima et al. observed similar relationships between BCAAs and lipids in subjects without T2DM in a cross-sectional study of the Asian population [66]. We also found negative weak correlations of total BCAAs with TC and LDL-C. The data on TC and LDL-C association with BCAA concentrations are inconsistent. One study found positive weak correlations and another a lack of such a relationship [11,76,77]. Therefore, both our results and the results of other authors suggest that an increased concentration of BCAAs may be associated with dyslipidemia. The relationship between dyslipidemia and BCAAs may be explained by several potential mechanisms. The products of BCAA catabolism are involved in the biosynthesis of lipids. Leucine can be degraded to 3-hydroxy-3-methylglutaryl-CoA, which is one of the intermediates of the cholesterol synthesis pathway [78,79]. There is also a hypothesis that an increase in isoleucine degradation is associated with the accumulation of odd chain fatty acids (propionyl-CoA, a product of isoleucine degradation, might be a substrate for odd chain fatty acid synthesis) [78]. It was also reported that leucine in the mTOR signaling pathway promotes lipid synthesis by activating the sterol regulatory element-binding transcription factor-1, which is a master transcriptional regulator of insulin-stimulated fatty acid synthesis [80,81,82]. Additionally, high concentrations of BCAAs impair glucose metabolism and are positively associated with NAFLD risk, which impairs lipid homeostasis leading to upregulation of hepatic de novo lipogenesis and accumulation of triglycerides [83,84].

We also demonstrated a positive correlation between BCAAs and FLI values which was independent of glycemic status but became significantly attenuated after adjusting for BMI and age. We observed a similar tendency for a relationship between CRP and BCAAs. The results of our logistic regression and ROC analyses showed that these relationships were even stronger in the normoglycemic than the dysglycemic women. FLI is a simple algorithm based on waist circumference, BMI, TG, and GGT for the prediction of NAFLD risk [22]. Therefore, loss of statistical significance after adjustment for BMI may be explained simply by the fact that both these parameters, BCAAs and FLI values, are strongly related to BMI. The relationship between FLI and BCAAs has recently been observed by Berg et al. In this large-scale study among a Caucasian population without T2DM at baseline, FLI was related to total BCAAs independently of many factors, including insulin-resistance and β-cell function (the authors did not adjust for BMI) [84]. The pathophysiological mechanism of the relationship between liver dysfunction and BCAAs is complex, bidirectional, and linked to obesity and insulin resistance, and these relationships have been widely described by others [85,86,87,88,89]. Our results also suggest that the relationship between CRP and BCAA concentrations is independent of glycemic status and is mainly explained by BMI values. Most recently, Hamaya et al. showed a positive relationship between CRP and BCAAs which was independent of established CVD risk factors, including BMI, in a cross-sectional analysis of 19,472 women without a history of T2DM, CVD [90]. On the other hand, Sun et al. did not observe a relationship between BCAAs and CRP or Il-6 in a Chinese cohort (317 men and 294 women) [91]. In vitro studies showed that in cultured human peripheral blood mononuclear cells (PBMC), high BCAA concentrations promote oxidative stress from NADPH oxidase and mitochondria, the release of pro-inflammatory cytokines mediated by the activation of the nuclear transcription factor-κB (NF-κB), and the migration of PBMC via the activation of the mammalian target of rapamycin (mTORC1) axis [50]. Taken together, it seems that obesity related chronic diseases like insulin-resistance, NAFLD, inflammation, and dyslipidemia are linked to elevated circulating BCAAs, and these relationships may be explained by different mechanisms and, what is more, the cause-and-effect relationship is complex and not fully understood.

Our study has some limitations. The first limitation is the small size of the study groups. For this reason, we could not perform multifactor adjustments of our analyses. The second is the use of the enzymatic method for BCAAs, which measured only total BCAAs (as a sum of leucine, isoleucine, and valine). As was previously observed, the correlation between total BCAA concentrations measured by HPLC and by enzymatic analysis was excellent [92]. The enzymatic method for BCAAs is easy to perform, fast, and relatively cheap and does not require any highly specialized equipment, so it would be used in most clinical laboratories. Moreover, most previous studies have shown that the concentration of total BCAAs is associated with metabolic factors to a similar extent as individual BCAAs or even that this relationship is stronger [12]. Another limitation of our study is the lack of measurement of parathyroid hormone (PTH), phosphorus, and vitamin D, which are involved in the regulation of circulating calcium concentration. However, it was observed that an association between elevated serum calcium concentration and glucose, insulin resistance, or T2DM remained significant even after adjusting for these parameters [63]. Moreover, our study is not able to establish the biological mechanisms or causality responsible for the relationship between circulating calcium and BCAAs. We only showed the statistical relationships between these parameters and suggested some potential mechanisms based on the literature review.

## 5. Conclusions

In conclusion, total BCAA concentrations are associated with dysglycemia, dyslipidemia, inflammation, and NAFLD risk. The dysglycemic state in particular strongly predisposes women to significant positive relationships between total BCAAs and circulating calcium and HbA1c independent of BMI, and these relationships may potentially reflect the pathophysiological calcium-dependent mechanisms connecting BCAAs with metabolic disturbances.

## Figures and Tables

**Table 1:** Clinical characteristics of the women.

Parameters	Women with Normoglycemia *n* = 184	Women with Dysglycemia *n* = 165	*p*
BCAAs [µmol/L]	433.0 (407.2–493.8)	545.0 (468.1–620.9) $	<0.0001
Age [years]	48.4 ± 6.0	51.2 ± 5.5	<0.0001
BMI [kg/m^2^]	24.1 (21.9–28.4)	31.2 (25.8–37.7)	<0.0001
WC [cm]	82.5 (76.5–91.5)	98.0 (87.0–110.0)	<0.0001
Glucose [mmol/L]	5.1 (4.8–5.2)	5.8 (5.4–6.9)	<0.0001
TC [mmol/L]	5.6 ± 1.0	5.5 ± 1.2	0.18
LDL-C [mmol/L]	3.5 ± 0.9	3.4 ± 1.0	0.06
HDL-C [mmol/L]	1.6 (1.4–1.9)	1.4 (1.2–1.7)	<0.0001
TG [mmol/L]	1.0 (0.8–1.3)	1.5 (1.1–2.0)	<0.0001
TG/HDL-C	3.4 (3.0–4.0)	3.7 (3.3–4.5)	<0.0001
Creatinine [mg/dL]	0.76 ± 0.11	0.78 ± 0.12	0.11
eGFR [ml/min/1.73 m^2^]	94.1 (84.8–101.7)	89.6 (78.5–100.4)	0.008
CRP [mg/L]	0.82 (0.33–1.76)	1.70 (0.77–4.23)	<0.0001
HbA1c [mmol/mol]	36.0 (33.0–37.0)	39.0 (36.0–44.5)	<0.0001
TSH [mIU/L]	1.4 (1.1–2.0)	1.4 (1.0–2.1)	0.8
Insulin [µIU/mL]	5.1 (3.7–6.9)	10.0 (6.3–13.0)	<0.0001
HOMA-IR	1.11 (0.79–1.56)	2.6 (1.9–3.6)	<0.0001
TCa [mmol/L]	2.32 (2.26–2.40)	2.40 (2.30–2.51)	<0.0001
CCa [mmol/L]	2.24 (2.18–2.32)	2.33 (2.22–2.44)	<0.0001
Albumin [g/L]	44.0 (42.9–45.6)	43.7 (42.4–45.9)	0.40
FLI	16.0 (8.1–37.7)	64.4 (30.4–93.5)	<0.0001
GGT [U/L]	17.4 (13.8–21.9)	22.4 (16.4–33.3)	<0.0001
SBP [mmHg]	118 (109–130)	130 (120–140)	<0.0001
DBP [mmHg]	78 (71–84)	80 (78–88)	<0.0001
Postmenopausal status [%]	38	56	0.0004
Lipid lowering therapy [%]	11	28	0.0001
Hypertension treatment [%]	13	45	<0.0001
Diabetes [%]	0	43	<0.0001
Obesity [BMI ≥ 30%]	30	70	<0.0001
Current smoker [%]	17	20	0.47
Physically active: never or sporadically [%]	31	32	0.84

Notes: Means ±SD or medians (25th and 75th percentiles) or percentage; WC—waist circumference; SBP—Systolic blood pressure; DBP—Diastolic blood pressure; eGFR—estimated glomerular filtration rate (CKD-EPI); FLI—Fatty Liver Index; $ Difference in BCAA concentration was still significant after adjusting for BMI, age and menopausal status (*p* < 0.0001); CCa—albumin-corrected calcium; TCa—total calcium.

**Table 2:** Unadjusted and adjusted correlation coefficients between BCAAs and cardiometabolic risk factors.

Parameters	Total Group r0/r0 *	Women with Normoglycemia r1/r1 *	Women with Dysglycemia r2/r2 *	*p* Unadjusted r1 vs. r2
Age	0.24/-	0.17/-	0.25/-	ns
BMI	0.61/-	0.49/-	0.54/-	ns
TCa	0.37/0.17	0.05 ^ns^/-	0.46/0.31	<0.0001
CCa	0.47/0.21	0.23/0.06 ^ns^	0.51/0.33	0.002
HbA1c	0.47/0.27	0.12 ^ns^	0.41/0.30	0.004
HOMA-IR	0.41/0.18	0.07 ^ns^	0.27/0.16	0.05
Glucose	0.40/0.32	0.10 ^ns^	0.30/0.26	0.05
TC	−0.22/−0.13	−0.17/−0.18	−0.22/−0.09 ^ns^	ns
LDL-C	−0.20/−0.09 ^ns^	−0.12 ^ns^/-	−0.19/−0.07 ^ns^	ns
HDL-C	−0.46/−0.20	−0.33/−0.17	−0.46/−0.26	ns
TG	0.38/0.14	0.19/0.07 ^ns^	0.33/0.14 ^ns^	ns
TG/HDL-C	0.47/0.20	0.28/0.12 ^ns^	0.43/0.21	ns
TC/HDL-C	0.28/0.10 ^ns^	0.18/0.04 ^ns^	0.28/0.15 ^ns^	ns
eGFR	−0.29/−011 ^ns^	−0.28/−0.22	−0.23/−0.02 ^ns^	ns
FLI	0.57/0.10 ^ns^	0.49/0.008 ^ns^	0.48/0.10 ^ns^	ns
CRP	0.44/0.08 ^ns^	0.36/0.17	0.35/0.04 ^ns^	ns
SBP	0.18/0.004 ^ns^	0.06 ^ns^/-	0.08 ^ns^/-	ns
DBP	0.07 ^ns^/-	−0.02 ^ns^/-	−0.09 ^ns^/-	ns

Notes: r—correlation coefficient; All correlations were statistically significant *p* < 0.05 except for correlations flagged with ns; ns—not significant; CCa—albumin-corrected calcium; TCa—total calcium; r0, r1, r2—unadjusted correlation coefficients; r0 *, r1 *, r2 * correlation coefficients adjusted for age and BMI.

**Table 3:** Associations of BCAAs with cardiometabolic risk factors in logistic regression models.

Cardiometabolic Risk Factor	Total Group	NG Group	DG Group	Total Group	NG Group	DG Group
	Unadjusted ORs (95%CI) per10 unit increase in BCAAs			Adjusted for age and BMI ORs (95%CI) per10 unit increase in BCAAs		
CCa > 2.38 [mmol/L]	1.15 (1.11–1.19) $	1.11 (1.04–1.18) &	1.16 (1.10–1.22) $	1.09 (1.05–1.14) $	1.03 (0.96–1.11)	1.13 (1.07–1.19) $
TCa > 2.45 [mmol/L]	1.11 (1.08–1.15) $	1.07 (1.01–1.13) *	1.12 (1.07–1.17) $	1.07 (1.03–1.11) &	1.01 (0.94–1.07)	1.10 (1.05–1.15) $
HbA1c > 39 [mmol/mol]	1.10 (1.07–1.13) $	1.03 (0.97–1.10)	1.11 (1.06–1.15) $	1.10 (1.06–1.15) $	1.03 (0.95–1.10)	1.09 (1.04–1.14) &
FLI > 60	1.17 (1.13–1.21) $	1.19 (1.15–1.25) $	1.13 (1.08–1.17) $	1.04 (0.97–1.09)	1.06 (0.95–1.15)	0.98 (0.90–1.04)
eGFR < 90 mL/min/1.73 m^2^	1.06 (1.03–1.09) $	1.09 (1.04–1.15) &	1.06 (1.03–1.09) *	1.04 (1.0–1.07) *	1.08 (1.02–1.15) *	1.02 (0.97–1.06)
TG > 1.7 [mmol/L]	1.06 (1.04–1.09) $	1.03 (0.98–1.09)	1.04 (1.07–1.08) *	1.04 (1.0–1.07) *	0.98 (0.92–1.04)	1.03 (0.99–1.07)
HDL < 1.2 [mmol/L]	1.09 (1.06–1.12) $	1.09 (1,04–1.16) &	1.07 (1.03–1.12) *	1.05 (1.01–1.08) &	1.04 (0.97–1.12)	1.05 (1.10–1.09) *
CRP > 3.0 [mg/L]	1.10 (1.07–1.13) $	1.12 (1.05–1.20) &	1.07 (1.03-1.11) &	1.0 (0.96–1.05)	0.90 (0.91–1.07)	0.99 (0.95–1.04)
TC/HDL-C > 4.5	1.05 (1.02–1.07) &	1.03 (0.98–1.08)	1.02 (0.99–1.06)	1.01 (0.98–1.04)	0.97 (0.92–1.0)	1.01 (0.97–1.05)
Hypertension or therapy	1.07 (1.04–1.10) $	1.03 (1.0–1.08)	1.04 (1.0–1.10) *	1.02 (0.98–1.05)	0.98 (0.92–1.05)	0.99 (0.96–1.04)

Notes: ORs—odd ratios, CI—confidence interval; * *p* < 0.05; & *p* < 0.001; $ *p* < 0.0001; CCa—albumin-corrected calcium; TCa—total calcium; eGFR—estimated glomerular filtration rate; FLI—Fatty Liver Index; NG—normoglycemic group, DG—dysglycemic group.

**Table 4:** AUC values for associations between BCAAs and cardiometabolic risk factors according to glycemic status.

Cardiometabolic Risk Factor	Total Group AUC (95% CI)	NG Group AUC (95% CI)	DG Group AUC (95% CI)	*p* NG vs. DG
CCa > 2.38 mmol/L	0.81 (0.76–0.85)	0.67 (0.59–0.74)	0.83 (0.76–0.89)	0.043
TCa > 2.45 mmol/L	0.75 (0.69–0.79)	0.61 (0.54–0.69)	0.77 (0.69–0.83)	0.048
HbA1c > 39 mmol/mol	0.76 (0.70–0.80)	0.60 (0.52–0.68)	0.73 (0.63–0.78)	0.115
FLI > 60	0.84 (0.79–0.88)	0.85 (0.79–0.91)	0.75 (0.68–0.82)	0.111
eGFR < 90 mL/min/1.73 m^2^	0.65 (0.60–0.71)	0.67 (0.59–0.74)	0.65 (0.59–0.71)	0.621
TG > 1.7 mmol/L	0.69 (0.63–0.74)	0.57 (0.47–0.63)	0.63 (0.55–0.77)	0.273
HDL < 1.2 mmol/L	0.75 (0.69–0.79)	0.67 (0.59–0.74)	0.68 (0.60–0.75)	0.874
CRP > 3.0 mg/L	0.77 (0.72–0.81)	0.79 (0.71–0.84)	0.68 (0.60–0.76)	0.191
TC/HDL-C > 4.5	0.65 (0.59–0.70)	0.58 (0.49–0.65)	0.57 (0.50–0.66)	0.817
Hypertension or therapy	0.67 (0.61–0.72)	0.54 (0.46–0.62)	0.60 (0.51–0.67)	0.487

Notes: Area under the curve (AUC), confidence interval (CI), CCa—albumin-corrected calcium; TCa—total calcium; eGFR—estimated glomerular filtration rate; FLI—Fatty Liver Index; NG—normoglycemic group, DG—dysglycemic group.

## Data Availability

The data presented in this study are available on request from the corresponding author.
